# Rab8a/Rab11a regulate intercellular communications between neural cells via tunneling nanotubes

**DOI:** 10.1038/cddis.2016.441

**Published:** 2016-12-22

**Authors:** Hui Zhu, Chengbin Xue, Xi Xu, Yibing Guo, Xiaohong Li, Jingjing Lu, Shaoqing Ju, Yongjun Wang, Zheng Cao, Xiaosong Gu

**Affiliations:** 1 State Key Laboratory of Pharmaceutical Biotechnology and MOE Key Laboratory of Model Animal for Disease Study, Model Animal Research Center, Nanjing Biomedical Research Institute, Nanjing University, Nanjing, China; 2 Jiangsu Key Laboratory of Neuroregeneration, Co-innovation Center of Neuroregeneration, Nantong University, 19 Qixiu Road, Nantong, China; 3 Surgical Comprehensive Laboratory, Affiliated Hospital of Nantong University, 20 Xisi Road, Nantong, China; 4 Department of Rehabilitation Medicine, Affiliated Hospital of Nantong University, 20 Xisi Road, Nantong, China

## Abstract

Tunneling nanotubes (TNTs) are F-actin-based membrane tubes, and can form between cultured cells and within vital tissues. TNTs mediate intercellular communications that range from electrical signaling to the transfer of organelles. Following peripheral nerve injury, the orchestrated intercellular communications among neural and non-neural cells are required for effective nerve regeneration. It remains unknown whether TNTs exist between neural cells in the peripheral nerve system and how TNTs affect neural regeneration. To address these interesting questions, we investigated the transfer of neurotropic factors, membrane protein, cytoplasmic protein, mitochondria and RNA in functional TNTs formed between cultured Schwann cells (SCs). TNT-like structures were increased not only in cultured SCs after exposure to serum depletion but also in longitudinal sections of proximal sciatic nerve stump harvested after rat peripheral nerve transection. Meanwhile, downregulation of Rab8a or Rab11a in cultured SCs inhibited the formation of functional TNTs and vesicle transfer and led to decrease in cell migration, increase in SCs apoptosis. Likewise, knockdown of Rab8a or Rab11a in primary SCs also suppressed axonal outgrowth from co-cultured dorsal root ganglion (DRG) neurons. Overall, our results suggested that the gene of Rab8a or Rab11a might be involved in the formation of TNTs structures in the peripheral nerve system, while TNTs structures were likely to affect peripheral nerve regeneration through the regulation of neural cell communications.

Growing evidence for the intercellular transfer of molecules as large as proteins and cytoplasmic components through synapse, gap junctions, and tunneling nanotubes (TNTs) in aspect of tissue repair, immune response, cancer, normal tissue homeostasis and osteoclastogenesis has been reported.^1, 2, 3, 4, 5, 6^ As an nanoscaled, F-actin containing long membrane protrusions, TNTs facilitate the intercellular communication of diverse cellular signals and components ranging from electrical signaling to organelles.^5, 7, 8, 9^ Intercellular communication is related to many diseases and nanotubes are potentially useful as drug-delivery channels for cancer therapy. Although the occurrence of TNTs has been observed in many cell types *in vitro*, it remains to be determined whether the TNTs transfer mechanisms and their cargos are cell-type specific.^5^

In central nervous system (CNS), donor neurons overloaded with *α*-synuclein aggregates were recently reported as a mechanism of hijacking TNT-mediated intercellular trafficking to the neighbor cells which may contribute to the neuropathology—Parkinson's disease.^10^ Interestingly, the expression of mutant huntingtin (Htt) which increased the number of TNTs provided an efficient mechanism of transfer between neuronal cells to further illustrate the pathogenesis of Huntington's disease (HD).^11^

The communication and interactions between Schwann cells (SCs) and neurons are critical for the development and function of myelinated axons.^12^ Such interactions take place during development and in adulthood, and are critical for the homeostasis of the peripheral nervous system (PNS). Neurons provide essential signals to affect SCs function, whereas SCs promote neuronal survival and allow efficient transduction of action potentials. Deregulation of neuron-SC interactions often results in developmental abnormalities and diseases.^13^ Neurons and SCs exist in a highly interdependent relationship: damage to one cell type usually leads to pathophysiological changes in the other.^14^ Peripheral nerve injury (PNI) as a global clinical problem causes a devastating impact on patients' quality of life.^15, 16^ Although the PNS has a greater capacity of axonal regeneration than the CNS after injury, spontaneous repair of peripheral nerve is nearly always unsatisfied with poor functional recovery. Various types of medical therapy have been carried out for several hundred years with the intention of improving outcomes.^17^ Following PNI, orchestrated communication among neurons, SCs and other types of cells as fibroblasts and macrophages are required for effective nerve repair. Greater understanding of communication between these cell types will not only give insight into peripheral nerve development but also the new opportunities to enhance peripheral nerve regeneration.

Here, we found there were functional TNTs, in which the transfer of neurotropic factors, mitochondria and RNA were observed between cultured SCs and between neuron-SC either *in vitro* or *in vivo*. Downregulation of Rab8a or Rab11a in SCs reduced the formation of functional TNTs, decreased of vesicle transfer between the connected cells leading to SCs apoptosis and also suppressed axonal outgrowth from co-cultured dorsal root ganglion (DRG) neurons. Identification of the new mechanisms of neural cells communication contribute to our understanding of the development, physiology and pathology of the peripheral nervous system as a whole.

## Results

### TNTs between SCs and their properties

**Figure 1:**
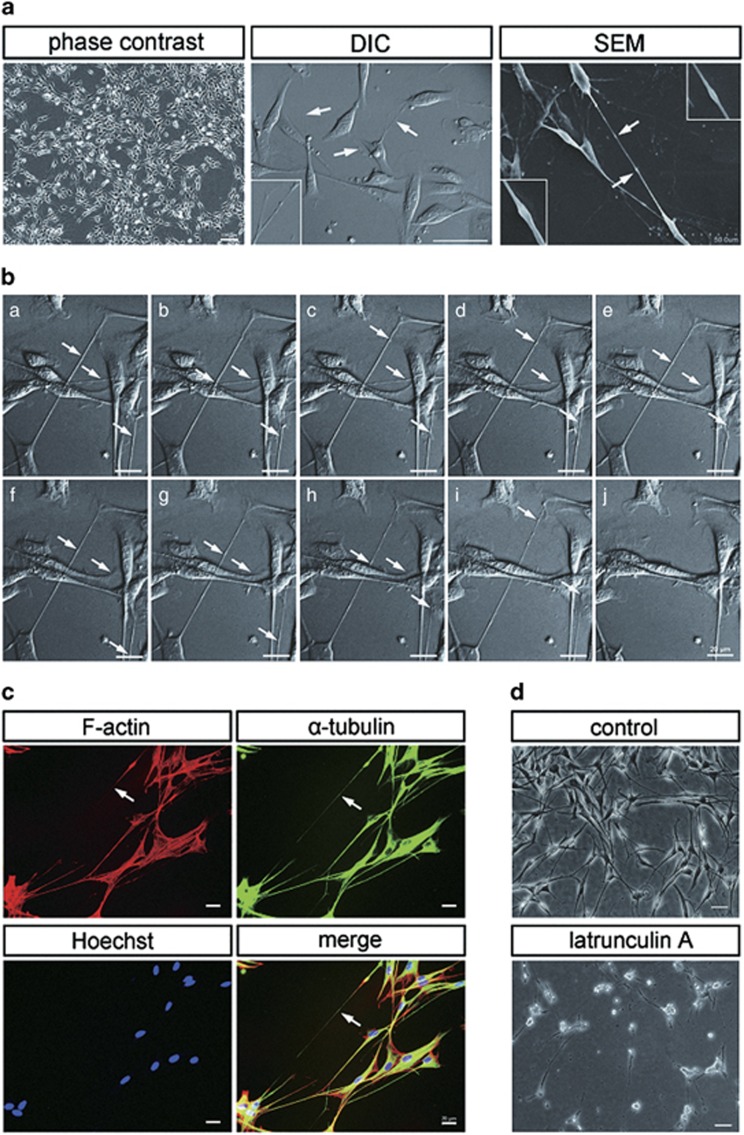
Characteristics and composition of TNTs in SCs. (**a**) TNTs were observed between SCs in semi-confluent cultures (left: phase contrast). The morphologies of TNTs were investigated by DIC microscope (center) and SEM (right). The arrow indicates a TNT. Higher magnification images are shown in boxed areas. TNTs with characteristic bulges represented transported cargo. (**b**) *De novo* formation of TNTs by time-lapse imaging. Selected frames of a video sequence (Supplementary Movies S1) are shown. The arrow indicates the object translocation. (**c**) TNTs contain both F-actin and a-tubulin. Fixed SCs were immunostained with an antibody against *α*-tubulin (green) and phalloidin-rhodamine (red), and hoechst (blue). (**d**) F-actin-disrupting agent latrunculin A-blocked TNT induction in SCs. Scale bars, 100 *μ*m, 20 *μ*m, 50 *μ*m (**a**); 20 *μ*m (**b**); 20 *μ*m(**c**); 50 *μ*m (**d**)

We initially observed TNTs hovering above the substratum and connecting primary SCs cultured *in vitro* (Figure 1a). The TNTs were highly resistant to trypsinization than other protrisions confirming previous observations.^4, 18^ Demonstration of the unique TNTs between primary SCs *in vitro* is provided in the accompanying Supplementary Movies Online (Supplementary Movies S1). TNTs formed within several hours of culturing cells once the cells began to adhere. Selected frames of a video sequence are shown (Figure 1b). When we performed scanning electron microscopic (SEM) analysis, the stretched shape and structure of TNTs could be preserved, and their surface showed a seamless transition to the surface of both connected cells. Unidirectional translocation of an object (arrowhead) along TNTs was observed (Figure 1a). Actin filaments, as the essential component of TNTs,^19^ were detected in TNT structures observed in culture of primary SCs by fluorescent F-actin marker (Figure 1c). This was further confirmed by the fact that adding F-actin-disrupting agent latrunculin A (100 nM) blocked TNT induction in SCs (Figure 1d). Similarly, we confirmed these TNT structures also contain *α*-tubulin, the cytoskeletal protein-forming microtubules (Figure 1c).

### TNTs facilitate intercellular transfer of lipophilic cytosolic components as well as proteins, mitochondria vesicles between SCs

**Figure 2:**
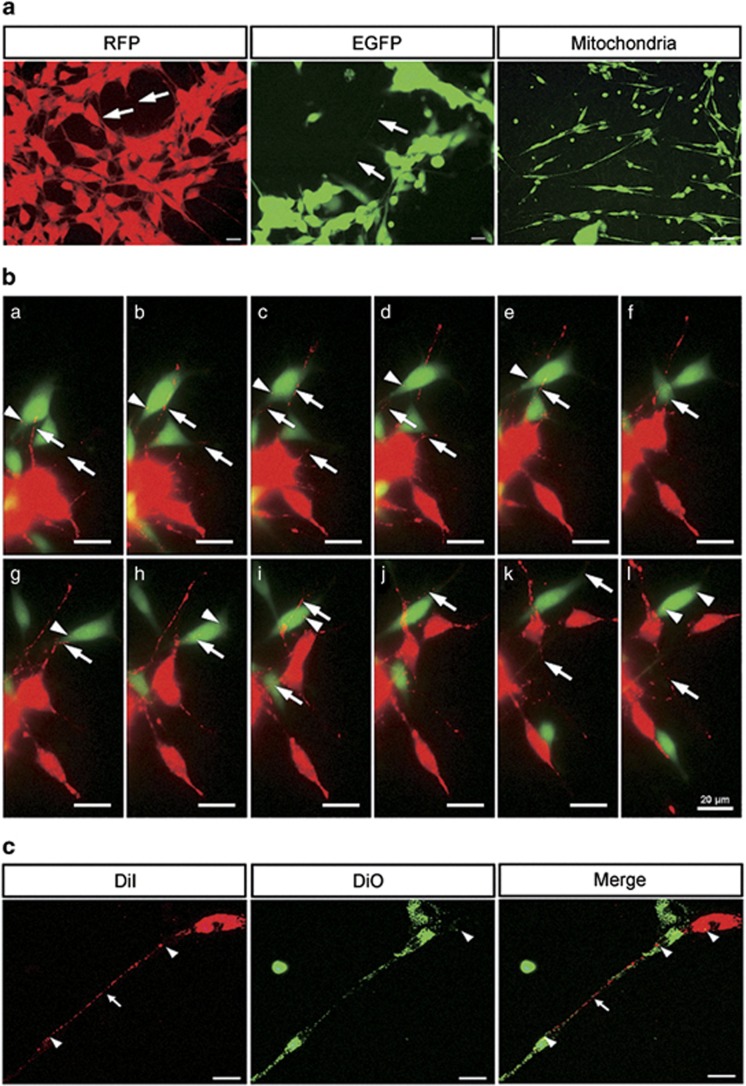
TNTs facilitate intercellular transfer of lipophilic cytosolic components as well as proteins, mitochondria vesicles between SCs. (**a**) SCs isolated from EGFP/RFP transgenic rats expressing formed TNTs which readily transmitted the EGFP or RFP between cells. TNTs also transfer mitochondria as well. (**b**) TNTs of co-cultured EGFP-SCs and RFP-SCs transmitted proteins via bidirectional transfer by time-lapse imaging. Selected frames of a video sequence (Supplementary Movies S2) are shown. (**c**) SCs stained with either green (DiO) or red (DiI) dyes formed TNTs, which transmitted lipophilic components when mixed. The arrow and arrowhead indicate the TNTs and transferred vesicles/ proteins, respectively. Scale bars: 20 *μ*m

To investigate whether cytoplasmic proteins could be exchanged between TNT-connected cells, we analyzed the transport of enhanced green fluorescent protein (EGFP) and red fluorescent protein (RFP) between two different cell populations. We observed that SCs isolated from newborn EGFP and RFP transgenic rats expressing EGFP or RFP formed TNTs which readily transmitted these proteins between SCs, and mitochondria as well (Figure 2a). In order to visualize transfer and determine identity of transmitted components, we co-cultured EGFP-SCs and RFP-SCs (quantity ratio 1 : 1) for 24 h and observed by time-lapse imaging (Figure 2b and Supplementary Movies S2). Some of them exhibited unidirectional transfer of lipophilic material. Like others, we also observed evidence of bidirectional transfer, in which red and green lipophilic components intermixed and transferred via TNTs, resulting in a yellowish or lighter color phenotype. To confirm these findings, we further used fluorophores such as the lipophilic dyes DiI (red fluorescence) and DiO (green fluorescence) to label cells and repeated the co-culture experiments. DiI and DiO labeled membrane proteins transferred via TNTs in both unidirectional and bidirectional fashion (Figure 2c).

### Newly synthesized RNA is transferred from SCs to axons after sciatic nerve transaction via TNTs

**Figure 3:**
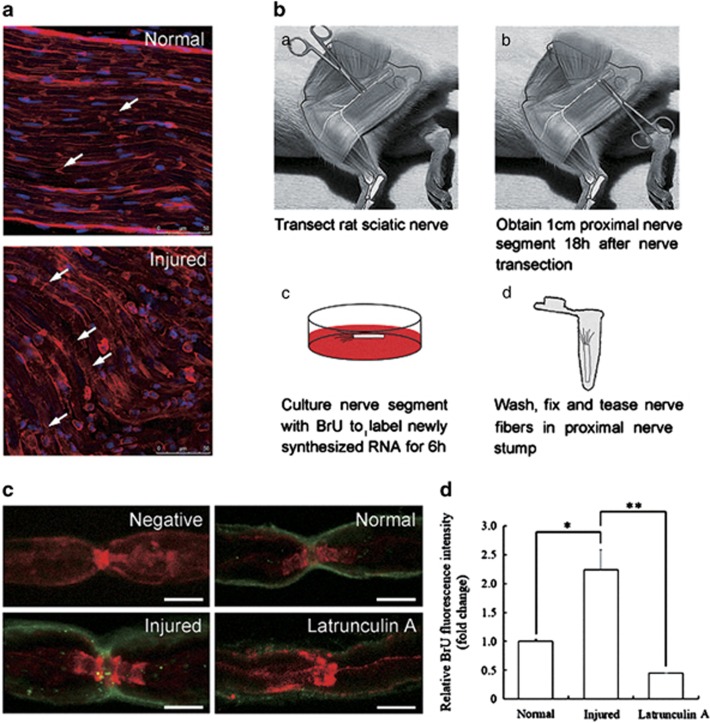
Newly synthesized RNA is transferred from SCs to axons after sciatic nerve transaction via TNTs. (**a**) Injured nerves showed increased formation of TNTs by F-actin staining. (**b**) Experimental procedure for BrU labeling. (**c**) Single confocal planes of fibers at nodes of Ranvier showing BrU incorporation (green) and F-actin (red). Negative control incubated with medium without BrU and 1.8 mg/ml latrunculin A during BrU labeling. Scale bars, 5 *μ*m. (**d**) Relative BrU fluorescence intensities for the normal, injured and latrunculin A treated nerves, representing RNA in the axon. (*n*=3) Data represented mean±S.E., *P<0.05 injured group compared with control groups. **P<0.01 latrunculin A group compared with injured groups

We hypothesized that TNTs are present not only in SCs cultured *in vitro* but also in rat sciatic nerve, which facilitate cargos transfer between SCs and axons to have a role in pathophysiological processes of PNI. To study whether the number of TNTs and the SCs-axons communications increase after sciatic nerve transaction, we detected the injured nerves and normal nerves with fluorescent phalloidin to label F-actin (red). Extensive fluorescent phalloidin was observed in proximal nerve stump compared with the normal nerve, and became reticular structure, which suggests there were increased formation of TNTs and more cargos transfer between SCs and axons (Figure 3a). Previously, it has been shown that cell-to-cell transfer of RNA is actin and myosin-Va dependent^20^ by labeling RNA with BrU. To further test the function of TNTs, we observed the transfer of newly synthesized RNA via TNTs after sciatic nerve transaction according to the optimized protocol (Figure 3b). The results showed increased SCs-to-axons RNA transfer but less in normal nerve. The most prominent labeling observed was a punctate labeling of axons at nodes of Ranvier (Figure 3c). The BrU signal significantly reduced when we applied latrunculin A during BrU labeling, which indicates the transfer is F-actin dependent. All these are consistent with the characters of TNTs *in vitro* (Figure 3d).

### Rab8a and Rab11a affect the formation of TNTs

**Figure 4:**
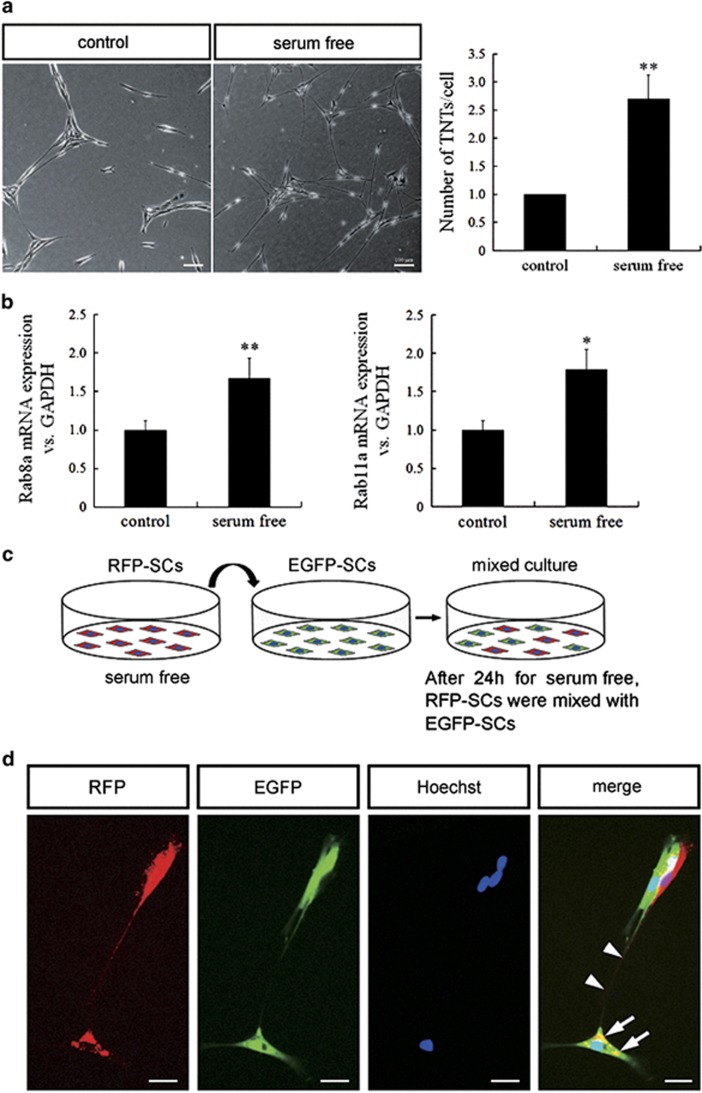
Serum depletion elicited more TNT formation and Rabs may be involved in TNT development. (**a**) More TNTs formed under serum-free medium compared with control group. Scale bars, 100 *μ*m. Quantification of TNT induction (*n*=3). Data represented mean±S.E., **P<0.01 compared with control groups. (**b**) Relative Rab8a and Rab11a mRNA expression level. *P<0.05 and **P<0.01 *versus* control. (**c**) Experiment procedure for co-culture of RFP and EGFP-expressing SCs. (**d**) When stress was applied to red cells, TNTs developed from RFP-SCs toward GFP-SCs. Scale bars, 20 *μ*m

Quantification of TNT induction showed serum depletion elicited more TNT formation compared with control group (Figure 4a). We also detected the mRNA expression level of the small GTPases Rab8a and Rab11a significantly increasing after the stress (Figure 4b), which were reported to have the important function in cell–cell communication. To address whether TNTs were induced by the target cells or they spouted out by the initiating cells, we distinguished SCs into two groups by transfecting EGFP or RFP, respectively. RFP-SCs were treated with TNT-inducing insults (serum depletion), and then trypsinized and washed, and then co-cultured with EGFP-SCs (Figure 4c). After induction by serum depletion, TNTs were developed between two groups of cells. We observed red TNTs developed from the stressed RFP-SCs to the green cells (Figure 4d)

### Endogenous Rab8a/11a is necessary for formation and function of TNTs

**Figure 5:**
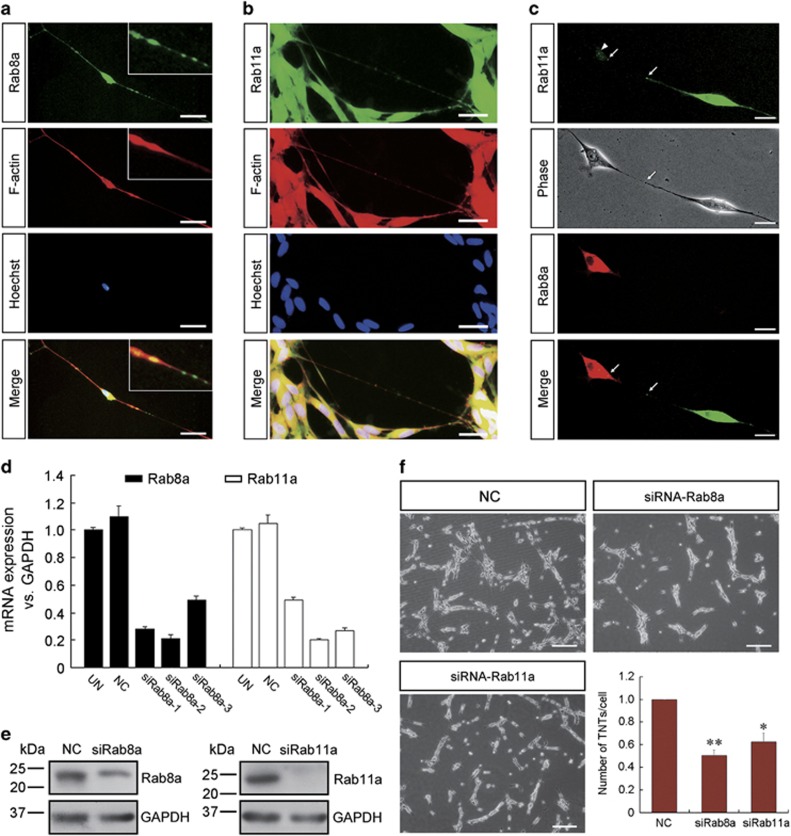
Rab8a and Rab11a are necessary for TNT formation in SCs. (**a** and **b**) Rab8a and Rab11a (green) co-localized with F-actin (red). Higher magnification images are shown in boxed areas. Scale bars, 20 *μ*m. (**c**) Transfected Rab8a-RFP and Rab11a-GFP also transferred between SCs via TNTs. Scale bars, 10 *μ*m. (**d**) qRT-PCR and (**e**) western blot analysis shown that the mRNA and protein expressions of Rab8a and Rab11a were significantly decreased by knockdown with siRNA-2. (**f**) Downregulation of Rab8a and Rab11a result in a decrease in the number of TNTs in SCs. *P<0.05 and **P<0.01 *versus* NC. Scale bars, 100 *μ*m

Both Rab8a and Rab11a were found in TNTs stained with phalloidin (Figure 5a and b). To investigate whether Rab8a/11a are linked to the formation of TNTs, SCs which were transfected with Rab8a-RFP or Rab11a-GFP transmitted fusion protein in TNTs via bidirectional transfer (Figure 5c). It is suggested that Rab8a/11a participate in the intercellular transfer involving TNTs. To further assess whether Rab8a/11a are required for TNT formation in SCs, we used siRNA to knockdown the endogenous levels of Rab8a and Rab11a expression (Figure 5d and e) and evaluated the effects on the number of TNTs between SCs. The number of TNTs decreased significantly when Rab8a/11a expression were downregulated (Figure 5f), indicating that Rab8a/11a are required for the formation of a subset of TNTs in SCs.

### Knockdown of Rab8a/Rab11a promote SCs apoptosis and inhibit migration via suppression of TNTs formation

**Figure 6:**
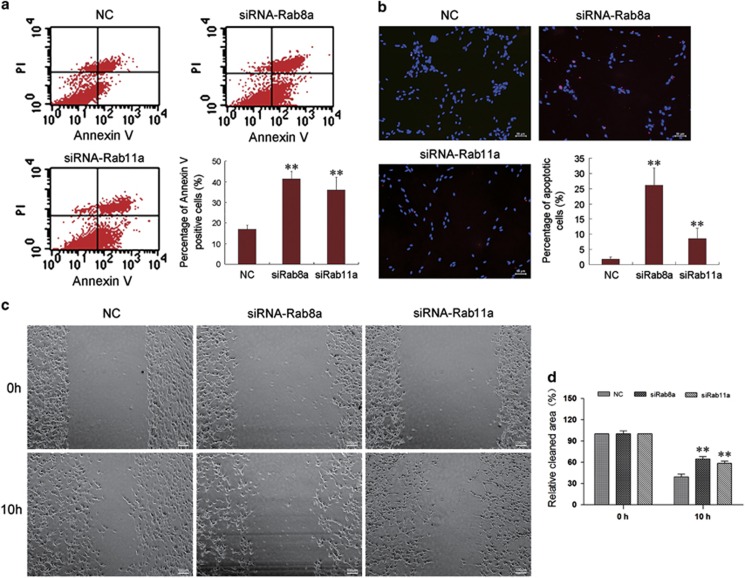
Knocking down Rab8a or Rab11a promote SCs apoptosis and inhibit SCs migration. (**a**) Flow cytometry showing the apoptosis rate of SCs after transfection with siRNA control (NC), Rab8a-siRNA (siRab8a) or Rab11a-siRNA (siRab11a). (**b**) TUNEL analysis showed increased apoptosis after SCs transfected with siRab8a or siRab11a. Scale bars, 50 *μ*m. (**c**) Wound-healing assay showed that transfection of siRab8a or siRab11a decreased SC migration compared with NC. Scale bars, 100 *μ*m. **P<0.01 *versus* NC

Knockdown of Rab8a/Rab11a promote SC apoptosis by annexin-V-FITC staining kit and TUNEL assay (Figure 6a and b). As TNTs can transfer mitochondria between adjacent cells, we hypothesized that Rab8a/11a may reduce the mitochondria transfer via suppression of TNTs formation, which lead to SCs apoptosis. Wound-healing assay indicated that transfection of SCs with Rab8a/11a significantly inhibited SC migration compared with that with negative control (NC) control (Figure 6c). It suggests that Rab8a/11a may regulate cell migration by linking actin dynamics to membrane turnover.

### Rab8a/Rab11a may affect neurotrophic factors transport and outgrowth of DRG neurons neurites co-cultured with SCs via TNTs

**Figure 7:**
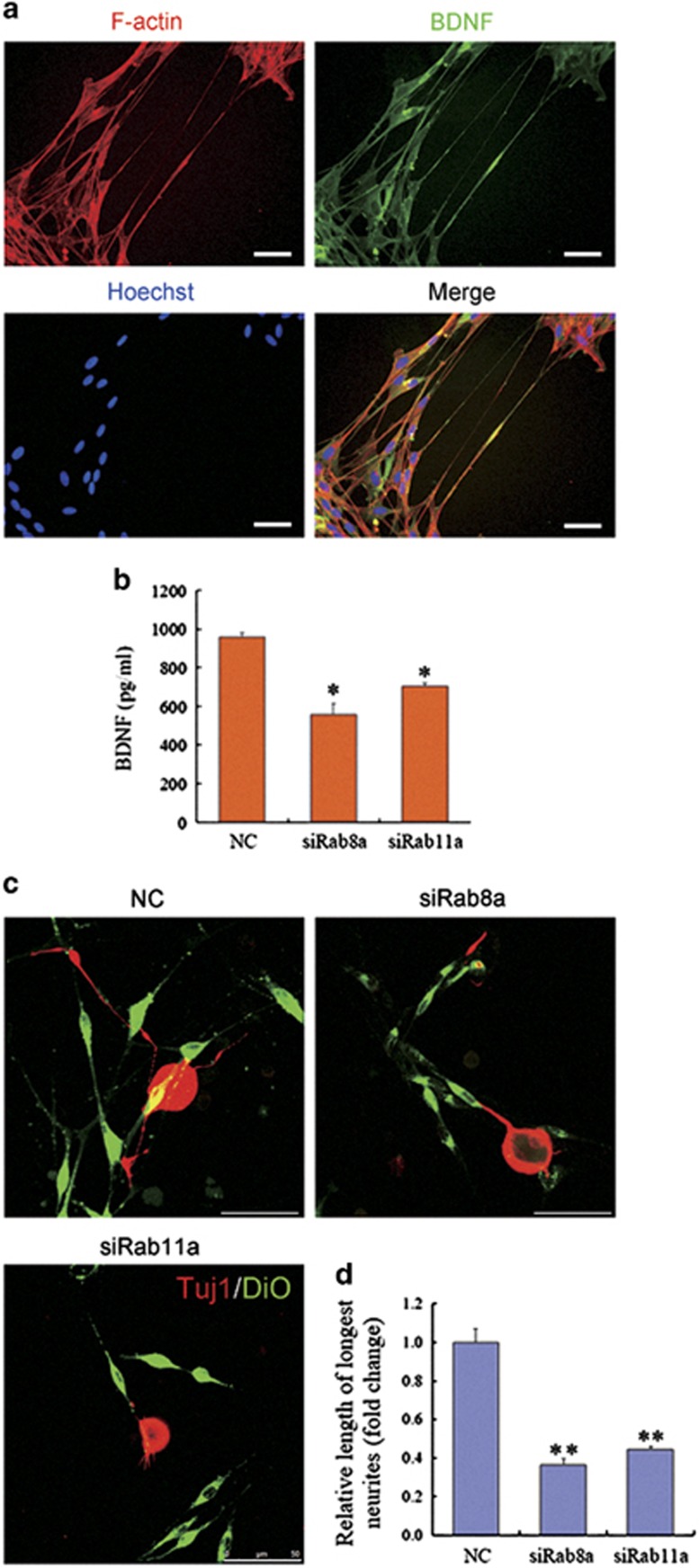
Effects of Rab8a or Rab11a on BDNF secretion of SCs and axonal outgrowth. (**a**) BDNF co-localiazation of F-actin indicates the transfer of BDNF may via TNTs. Scale bars, 50 *μ*m. (**b**) The ELISA data confirmed that BDNF secretion was reduced from SCs transfected with Rab8a or Rab11a siRNA. (**c**) Immunostaining with anti-Tuj1 showing that axon outgrowth was decreased in co-culture consisting of DiO labeled SCs, which had been transfected with Rab siRNA. Scale bars, 50 *μ*m. (**d**) Relative length of longest neurites was analyzed with Image-Pro Plus 7.0 software. *P<0.05 and **P<0.01 *versus* NC

Neurotrophic factors BDNF co-localization of F-actin indicates the transfer of BDNF may via TNTs (Figure 7a). BDNF secretion was reduced by SCs transfected with Rab8a or Rab11a siRNA (Figure 7b). Axonal outgrowth of DRG neurons was remarkably reduced co-cultured with SCs, which had been transfected with Rab8a/ Rab11a siRNA (Figure 7c and d). TNTs also reduced between SCs and DRG neurons by downregulating Rab8a/ Rab11a. It suggests that Rab8a/ Rab11a may affect both neurotrophic factors transport in SCs and outgrowth of neurites in co-cultured DRG neurons via TNTs.

## Discussion

Previous studies have shown that TNTs formation facilitates organelle transport between cultured cells.^4^ The membrane nanochannels can network various cells including immune cells, primary macrophages, neonatal rat cardiomyocytes, natural killer cells and hematopoietic stem cells.^21, 22^ Depending on the cell type, the TNTs formation can be activated by different mechanisms. The TNTs may be various in their cytoskeletal compositions, comprising either actin filaments alone or both actin filaments and microtubules.^23^ A comprehensive study of TNTs in various cell types will allow the classification of TNTs based on their specific functions, cargo and morphological features.

The most common approach to reveal whether a TNT belongs to a single cell or to both connected cells, is double-labeling with fluorescent dyes.^24, 25^ To visualize transfer and determine identity of transmitted components, we used fluorophores such as the lipophilic dyes DiI and DiO. However, this does not prove the direct intercellular transfer of material because such exchange might also happen through exocytosis or endocytosis. Here, the primary SCs isolated both from EGFP and RFP transgenic rats were also applied. We clearly saw the mixing of cytoplasm, and the transfer of cargo through the TNTs. TNTs have been shown to mediate the transfer of mitochondria between SCs (Figure 2a), supporting the notion that TNTs may channel organelle transfer. Furthermore, RFP, EGFP and cell membrane proteins transfer occurred in SCs, suggesting that this phenomenon represents a general mechanism of communication in cells.

TNTs development is a property of cells under stress, which can be induced in rat hippocampal astrocytes and neurons with H_2_O_2_ or serum depletion.^26^ TNTs formed spontaneously during *in vitro* growth in semi-confluent cultures, but were noted to be most prevalent in serum-free medium which is consistent with prior studies, in which serum depletion elicited TNTs formation in astrocytes under oxidative stress and a study which suggested that a low-serum, hyperglycemic, acidic growth medium can stimulate TNTs formation.^18^ Importantly, the number of cells per field was relatively constant and with significantly less proliferation of cells grown in the serum-free medium. Thus we demonstrated that an increase in numbers of TNTs was a reflection of an increase in *de novo* TNTs formation, and not due to an increase in cell numbers from proliferation. In our study, the result showed more TNTs formation in primary SCs cultured in serum-free medium than that in control group. Similar to the result *in vitro*, sciatic nerve transection accompanied by a series of pathological changes of ischemia and hypoxia in local nerve tissues, which elicited increased formation of TNTs and more cargos transfer between SCs and axons. The identification of specific TNTs structure *in vivo* is critical to confirm their role in the progression of myelination and axonal outgrowth after PNI.

In general, intercellular communication via TNTs represent a novel and not yet to be completely understood type of cell-to-cell interaction. Thus, a detailed understanding of the mechanisms of SCs communication might open new avenues for neural therapies. Here, we proposed that the number of TNTs between neural cells increases during SC activation (sciatic nerve transection), accompanied by the activation of related molecular signaling pathways which followed by increase of cell motility in neural regeneration. Migrating SCs form additional TNTs to preserve adherent junctions and intercellular communication. TNTs served as highways for signals of coordinated cell migration.

After nerve injury, axons in the proximal stump degenerate for some distance back from the injury sites. Meanwhile, inflammatory response is triggered and cell apoptosis is induced.^27^ As an important regulatory mechanism of tissue homeostasis, apoptosis triggered by the extrinsic pathway through the activation of pro-apoptotic receptors or by the intrinsic pathway through the destabilization of mitochondria in response to various forms of cell injury or stress.^28^ Our results of flow cytometry analysis and TUNEL assay indicated that Rab8a/Rab11a may have important roles in cell apoptosis by regulating the formation of TNTs. Both Rab8a and Rab11a are probably the potential target for regulating cell apoptosis.

Here, we directly demonstrated that TNTs formed in one type of neural cells can efficiently transfer to another in PNS. To characterize the transfer mechanism we had to distinguish between direct cell–cell transfer, which requires cell contact, and transfer through the medium. Transfer of mitochondria through TNTs has been shown in many cell types,^29, 30, 31^ where it enhances chemo-resistance. TNT-mediated transfer of functional mitochondria may reverse stressed cells in the early stages of apoptosis.^28^ It was reported that p53 and epidermal growth factor receptor are crucial for TNTs development. Akt, phosphoinositide 3-kinase and mTOR are involved in TNT induction.^26^ In our study, the TNTs connecting SCs contained Rab8a- and Rab11a-positive vesicles (Figure 5a). Rabs are a large family of small GTPases that regulate multiple steps in membrane traffic, including the uncoating, movement, maturation, tethering and fusion of vesicles with their target membranes. Multiple Rabs are associated with secretory granules/vesicles.^32^ Rab8 is a small GTPase that controls cell surface structures such as primary cilia, filopodia and neurites by regulating the actin cytoskeleton and the polarized delivery of receptors and vesicles to these specialized membrane domains.^33^ Both Rab8 and Rab11 has important roles in regulating cell migration.^34, 35^ Rab8 was also demonstrated present in TNTs and transferred between cancer cells.^36^ Forced expression of dominant negative Rab11 strongly impairs myelin formation *in vitro*.^37^

During nerve injury, SCs will return to an immature-like phenotype in the absence of axonal signals. However, SCs retain the ability to re-differentiate after injury, demonstrating remarkable plasticity throughout adult life. Although these cellular transformations are normally beneficial, during diseases in PNS, they are often disrupted to produce negative consequences. The co-culture model of SCs and DRG neurons *in vitro* indicated that Rab8a/Rab11a might regulate the axonal outgrowth via TNTs formation among these cells. It is beneficial to re-establish the connections by manual intervention of related molecular signaling pathways. The signaling molecules of Rab8a and Rab11a may not be the only way to intervene the formation of TNT-like structures in peripheral nerve system. The role of TNTs in intercellular communication after nerve injury is worthy to make further study.

## Materials and methods

### Primary SCs culture and oligonucleotide transfection

SCs were isolated from the sciatic nerves, as previously described.^38^ Briefly, sciatic nerves were harvested from Sprague–Dawley rats (1 day old) and enzymatically dissociated by incubation at 37 °C sequentially with 1% collagenase and 0.125% trypsin for 30 and 10 min, respectively. The mixture was triturated, centrifuged, and resuspended in Dulbecco's modified Eagle's medium (DMEM) supplemented with 10% (v/v) fetal calf serum. The cell pellets were plated on poly-l-lysine pre-coated dishes (35 mm) for incubation in the same medium. On the following day, 10 *μ*M cytosine arabinoside was added and allowed to incubate for additional 48 h to remove fibroblasts. The cell culture was maintained subsequently in DMEM supplemented with 10% FBS, 2 *μ*M forskolin (Sigma, St Louis, MO, USA), and 2 ng/ml heregulin (HRG, Sigma) to stimulate SC proliferation. For further purification, the cell culture was gently trypsinized, pelleted, and incubated with anti-Thy1.1 antibody (AbD Serotec, Raleigh, NC, USA) on ice for 2 h, followed by incubation in complement (Jackson Immuno, West Grove, PA, USA) for additional 1 h. All media and supplements were bought from Gibco-Invitrogen (Carlsbad, CA, USA). The final preparations consisted of 98% SCs, as determined by immunofluorescence for S100, which is a specific SCs marker. Primary culture of SCs were maintained in DMEM containing 10% fetal bovine serum (complete medium) at 37 °C under humidified 5% CO_2_. The culture of SCs were passaged no more than three times before conducting experiments. SCs were transfected with siRNAs (Ribobio, Guangzhou, China), respectively, using Lipofectamine RNAiMAX transfection reagent (Invitrogen), according to the manufacturer's instructions.

### Time-lapse imaging of live samples

Time-lapse imaging experiments were performed on an inverted time-lapse Olympus X181 Cell-R microscope equipped with a Hamamatsu CCD ORCA/AG camera and 960 or 9100 objective lenses. Cells were maintained at 37 °C, 5% CO_2_ in a humid environment. Procession of movies was made with Cell-R software (Olympus, Tokio, Japan). For determining timeframe of TNTs formation, bright field time-lapse images were taken every 15 min for 24 h. For all other time-lapse experiments, DIC (differential interference contrast) or phase contrast images were taken in addition to fluorescent imaging. Acquisition frequency was every 3 min for up to 3 h.

### Scanning electron microscopy

For SEM, SCs were fixed with 2.5% glutaraldehyde, dehydrated in graded series of ethanol and critical point dried using CO_2_. Afterwards cells were coated with gold using a JEOL JFC-110E Ion Sputter (JEOL, Tokyo, Japan) before observation under a Philips XL-30 scanning electron microscope (Eindhoven, The Netherlands).

### Quantitative real-time PCR

Reverse-transcribed complementary DNA was synthesized with the Prime-Script RT reagent Kit (TaKaRa, Dalian, China). PCR was performed with SYBR Premix Ex Taq (TaKaRa, Dalian, China). The relative expression level was calculated using the comparative 2^-△△Ct^ method. The sequences of Rab8a primer are as follows (5′-3′): forward, TTGGATTCGGAACATTGAGG; reverse, GCCTCTTGTCGTTCACATCAC. The sequences of Rab11a primer are as follows (5′-3′): forward, GCAACAAGAAGCATCCAGG; reverse, GCACCTACTGCTCCACGAT.

### Western blot analysis

Protein samples were extracted from cell cultures. Equal amount of protein samples were separated by SDS-PAGE, and transferred to PVDF membranes, which were blocked, and reacted with primary antibodies against Rab8a (1:1000, Abcam, Cambridge, UK), Rab11a (1 : 500, Abcam), or GAPDH (1:1000, Proteintech, Wuhan, China) according to the manufacturer's recommendations. The specific binding of primary antibody was detected by HRP-conjugated species-specific secondary antibody (Beyotime, Shanghai, China) and enhanced chemiluminescence assay.

### Sciatic nerve transection

Adult male SD rats, weighing 200–250 g, were anaesthetized by an intraperitoneal injection of 3% sodium pentobarbital solution (30 mg/kg body weight) before the sciatic nerve was exposed by making a skin incision and splitting the underlying muscles in the left lateral thigh. Then the sciatic nerve was transected and surgical incision rats was closed in a routine fashion. After 18 h, the rats were euthanized and a 2 cm sciatic nerve segment proximal to the transection was removed. Equivalent contralateral uninjured segments were used as NCs. The segments were incubated in Neurobasal medium (Invitrogen) containing 2.5 mm bromouridine (BrU,) for 6 h at 37 °C, 5% CO_2_. F-actin was depolymerized by the addition of 1.8 mg/ml latrunculin A (Sigma) during the BrU labeling step. In all experiments, segments were washed 10 times for 5 min each in ice-cold PBS buffer to remove unincorporated BrU, then fixed for 30 min in 4% paraformaldehyde in PBS at room temperature. Segments were treated for 1 h at 37 °C with 0.2 mg/ml collagenase (Sigma) in PBS. The nerve fibers were released from epineurium with #5 forceps and teased at the injured end with 26-gauge needles. The segments were permeabilized with 0.1% triton X-100 in PBS buffer for 30 min at room temperature.^20^

### Immunocytochemistry

Nerve segments were prepared for immunocytochemistry by blocking in 3% normal goat serum for 30 min at 37 °C. Permeabilized fibers were incubated with anti-BrU (Sigma, 1 : 300) overnight at 4 °C. Fibers were washed 6 times 5 min each. Secondary antibodies (goat anti-mouse or goat anti-rabbit conjugated with Alexa 488 (Abcam, 1 : 1000) were incubated for 45 min at 37 °C. F-actin was detected using fluorescent phalloidin (Invitrogen) added together with secondary antibodies. Fibers were then washed six times 5 min each. Finally, individual fibers were teased and mounted in ProLong Antifade (Invitrogen).

### Confocal microscopy

Teased fibers were visualized with a confocal laser scanning microscope (TCS SP2, Leica, Wetzlar, Germany). Images were processed with Image-Pro Plus 7.0 software (Media Cybernetics, Silver Spring, MD, USA). Nodes of Ranvier chosen for quantitative analysis were all within 100 mm of the injured end.

### TNT quantification

SCs were seeded at a density of 3 × 10^4^ cells in a 24-well plate for easily TNT quantification. To evaluate the number of TNTs as structures connecting two cells that do not touch the substratum, the number of all TNTs and total cells (labeled with Hoechst33342) were measured in each image using Image-Pro Plus 7.0 software (Media Cybernetics). For statistical analysis, 30 fields of images for each group collected from three independent experiments and then the number of TNTs per cell were calculated to ensure consistency and reproducibility.

### Evaluation of cell apoptosis by flow cytometry analysis

Cells were harvested using 0.25% trypsin and then washed with 0.1 M PBS. After centrifugation at 800 × g for 5 min, cells were treated with 100 *μ*l binding buffer and then stained with 2 *μ*l propidium iodide and 2 *μ*l annexin V-fluorescein isothiocyanate for 15 min. In this study, an annexin-V-FITC staining kit (Roche Applied Science) was used to assess cell apoptosis, we used the BD FACS Calibur flow cytometry (BD Bioscience, San Jose, CA) and the MACSQuant flow cytometry (Miltenyi Biotechnology, Bergisch Gladbach, Germany) to examine the cell apoptosis. Three independent flow cytometric experiments were performed.

### TUNEL staining

Cells were fixed in fresh 4% paraformaldehyde and 4% sucrose in PBS for 20 min at room temperature and permeablized in 0.1% Triton X-100 and 0.1% sodium citrate in PBS for 2 min on ice. Terminal deoxynucleotidyl transferase-biotin dUTP nick-end labeling (TUNEL) staining was performed using the *in situ* cell death detection kit I as described by the manufacturer (Roche, Basel, Switzerland). The coverslips were washed once in PBS for 5 min and then mounted on glass slides to be observed under a fluorescence microscope. Data were expressed as the ratio of apoptotic cells to total cells.

### Wound-healing assay of primary SCs *in vitro*

SCs transfected with siRNA were seeded on a 24-well plate and allowed to incubate for 24 h to form a tight cell monolayer, followed by starvation in DMEM supplemented with 0.5% fetal bovine serum (Invitrogen) and 0.15 *μ*g/ml mitomycin C (Sigma) for 12 h. Afterward, the cell monolayer was wounded with a 200 *μ*l plastic pipette tip. The remaining cells were washed with the above medium to remove cell debris and allowed to incubate at 37 °C in the low-serum containing culture medium for 10 h. Then, migrating cells at the wound front were photographed, and the cleaned area at each time point (the percentage relative to that at 0 h) was measured using Image-Pro Plus software (Media Cybernetics).

### Enzyme-linked immunosorbent assay (ELISA)

Primary SCs were transfected with Rab8a/Rab11a siRNA and control, respectively, using Lipofectamine RNAiMAX transfection reagent (Invitrogen). After incubation for 24 h, the medium of transfected SCs was replaced with FBS-free medium for addition 48 h incubation. The medium was then taken out and filtered through a 0.22 *μ*m filter (Millipore, Bedford, MA, USA) to furnish the supernatant. The protein levels of BDNF in the medium were measured using a ChemiKine BDNF ELISA Kit (Millipore) according to the manufacturer's instructions. Data were measured and summarized from 3 independent experiments, each comprising triplicate wells.

### Co-culture of DRG neurons and SCs

Primary SCs were transfected with siRNA and control respectively, by the above described protocols, and then co-cultured with rat adult DRG neurons, which had been prepared as described previously^39^ and did not undergo any transfection. After 24 h co-culture of SCs with DRG neurons, and fixed with 4% paraformaldehyde to undergo immunocytochemistry with anti-Tuj1 antibody (Sigma) to observe axon outgrowth. The longest neurite length for each of the neurons with processes longer than one cell bodies in diameter was measured with Image-Pro Plus software. The mean and S.E.M. of neurite-bearing cells were calculated from at least three independent experiments.

### Statistical analysis

The data are expressed as the mean±S.E.M. For statistical analyses, the data were replicated in at least three experiments. Statistical significance between two groups was calculated using either a two-tailed Student's *t*-test or one-way ANOVA followed by Bonferroni *post hoc t*-test. Differences were considered significant at *P*<0.05 (*) and *P*<0.01 (**).
